# The interaction of feeding an eubiotic blend of essential oils plus 25-hydroxy-vit-D3 on performance, carcass characteristics, and dietary energetics of calf-fed Holstein steers

**DOI:** 10.3389/fvets.2022.1032532

**Published:** 2022-12-01

**Authors:** Brooke C. Latack, Pedro H. V. Carvalho, Richard A. Zinn

**Affiliations:** ^1^Cooperative Extension, Division of Agriculture and Natural Resources, University of California, Holtville, Holtville, CA, United States; ^2^Department of Animal Science, University of California, Davis, Davis, CA, United States

**Keywords:** essential oil, monensin, cattle, feedlot, Holstein

## Abstract

Bans on the use of ionophores in several regions of the world has led to a need to identify alternative feed additivies to be added in cattle diets. Essential oil blends have been identified as a potential alternative to ionophores in feedlot diets. The objective of this study was to evaluate the effects of a supplemental a blend of essential oils and 25-hydroxyvitamin D3 on growth performance, energetic efficiency, and carcass characteristics in calf-fed Holstein steers. Ninety Holstein steer calves (123 ± 7 kg; 4 months old) were randomly assigned to 18 pens (5 steers/pen; 6 pens/treatment). Dietary treatments consisted of a steam-flaked corn-based diet supplemented with (DM basis): (1) no additives (CON); (2) 30 mg/kg DM of monensin (MON); (3) 200 mg/kg DM of a mixture of essential oils plus 25-hydroxyvitamin D3 (EO+HYD). There were no treatment effects (*P* > 0.05) on initial, intermediate and final cattle live weight; moreover, cattle had similar (*P* > 0.05) average daily gain (ADG) and dry matter intake (DMI) among dietary treatments. However, during the first 112 days of feed, calf-fed Holstein steers supplemented with EO+HYD had a greater (*P* ≤ 0.05) gain to feed ratio (G/F) than cattle fed the control diet but similar (*P* > 0.05) G/F to cattle supplemented with MON. However, there was no effect (*P* > 0.05) of dietary treatments on 112 to 286 d and the overall G/F ratio of calf-fed Holstein steers. Calf-fed Holstein steers supplemented with EO+HYD had greater (*P* ≤ 0.05) estimated net energy for maintenance (NEm) and net energy for gain (NEg) based on cattle growth performance than cattle fed the CON diet. Cattle supplemented with MON had an intermediate and similar (*P* > 0.05) NEm and NEg compared to the other two dietary treatments. However, when observed vs. expected NEm and NEg were calculated, cattle supplemented with MON and EO+HYD had greater efficiency of dietary energy utilization than cattle fed the CON diet. Calf-fed Holstein steers supplemented with MON had greater (*P* < 0.05) fat thickness than EO+HYD supplemented steers, and both were intermediate (*P* ≥ 0.05) to that of cattle fed the CON diet. There were no other effects (*P* > 0.05) on kidney, pelvic and heart fat, longissimus area, marbling score, and retail yield. The health status of cattle and liver abscesses or liver scars at slaughter were similar (*P* > 0.05). We conclude that supplementing calf-fed Holstein steers with MON or EO+HYD for over 285 days increased dietary net energy utilization for maintenance and gain of the diet by 3 and 4%, respectively, compared to non-supplemented steers.

## Introduction

Monensin (MON) is among the most commonly used ionophores in the growing-finishing diets for feedlot cattle (1). Enhancements in gain efficiency and digestive function (2) are partially attributable to selective action on the gram-positive bacteria in the rumen, potentially decreasing ruminal acetate:propionate molar ratio and methane energy losses (3, 4), and reduction in maintenance energy requirements (5). However, the present ban on ionophore supplementation within the European Union (6), and the potential trend elsewhere, have led to the quest for alternatives to conventional antibiotic supplements. Among those alternatives are plant-extracted essential oils. As with the ionophores, essential oil (EO) supplementation may reduce acetate:propionate molar ratio, methane production, and ruminal protein degradation (7). Moreover, recent studies have reported that growing-finishing cattle fed high energy diets supplemented with EO or MON had similar growth performance and carcass characteristics effects (8–10). However, the broader antimicrobial activities of EO within the rumen (7, 11, 12) may also lead to negative responses in organic matter digestion and cattle growth performance.

Another alternative feed additive that has been recently explored in the literature is the supplementation of 25-hydroxy-vitamin-D3 (HYD).

It has been reported that cattle supplementation with HYD increased average daily gain (13), carcass weight (14), and dressing percentage (15) compared to cattle receiving a diet without supplementation of HYD. However, these studies did not aimed to include an EO plus HYD in their dietary treatments. Crina^®^ Ruminants is a eubiotic blend composed of thymol, eugenol, limonene, and vanillin, while HyD^®^ is the 25-hydroxyvitamin D3, an active metabolite of vitamin D3 (DSM Nutritional Products, El Salto, Jalisco, MX) for 286 days. Therefore, the effect of the combination of EO + HYD on cattle growth performance and carcass characteristics of calf-fed Holstein steers that stay on feed for long periods is still lacking.

According to the latest National Beef Quality Assurance Audit, the percent of Holstein steers fed for slaughter in the United States increased around 60% from 2011 to 2016 (16, 17). In 2016, Holsteins represented 16% of total cattle fed for slaughter in the U.S. (17). Calf-fed Holstein steers enter the feedlot at characteristically light weights (115–180 kg) represents most of the cattle fed in southwest desert region in the U.S., where they are fed for periods typically over 285 days (18, 19). However, studies evaluating the effects of EO + HYD supplementation on the growth performance of calf-fed Holstein steers fed grain-based diets over 285 days are limited. Therefore, the objective of this study was to evaluate the interaction of supplemental Crina^®^ Ruminants and HyD^®^ (EO+HYD) on growth performance, energetic efficiency, and carcass characteristics in calf-fed Holstein steers fed a conventional growing-finishing diet.

## Materials and methods

All procedures involving animal care and management were in accordance with and approved by the University of California, Davis, Animal Use and Care Committee (protocol # 18811 and 18812).

Ninety Holstein steer calves (123 ±7 kg; 4 months old) originating from Tulare, California, were received at the University of California Desert Research Center, Holtville, CA. Upon arrival, calves were vaccinated for IBR, BVD, PI_3_, and B.R.S.V. (Bovi-shield^®^ Gold 4, Zoetis Animal Health, New York, NY), clostridial (UltraChoice^®^ 8, Zoetis Animal Health, New York, NY), gram-negative septicemic diseases (Endovac-Beef; IMMVAC, Inc. Columbia, MO), treated for parasites (Dectomax^®^ Injectable, Zoetis Animal Health, New York, NY) and injected (S.C.) with 400 mg Tulathromycin (Draxxin, Zoetis, Kalamazoo, MI). Steers were randomly assigned to 18 pens (5 steers/pen). Pens were 79 m^2^ with 27 m^2^ overhead shade, automatic waterers and 4.22 m fence-line feed bunks. During the initial 28-d adjustment period, all calves were fed the same non-supplemented (control) basal diet. On d 28, calves received Endovac-Beef and Ultra Choice 8 booster vaccinations, injected with 1,000,000 IU vitamin A (Vitamin AD, Huvepharma, Inc., St. Joseph, MO), weighed, and the trial was initiated. Dietary treatments are shown in Table 1, consisting of a steam-flaked corn-based diet supplemented with (DM basis): (1) no additives; (2) 30 mg/kg DM of monensin; (3) 200 mg/kg DM of a mixture of Crina^®^ (dosage 0.1g/kg DM) plus HyD^®^ (dosage 0.1 mg/kg DM). Diets were prepared weekly and stored in plywood boxes in front of each pen. Steers were allowed *ad libitum* access to water and dietary treatments. Fresh feed was provided daily. On days 112 and 224, all steers were injected subcutaneously with 500,000 IU vitamin A (Vital E-A + D, Stuart Products, Bedford, TX) and implanted with Revalor-S (Intervet, Millsboro, DE). The health status of cattle was monitored daily by trained personnel for signs of illness or pinkeye. Cattle with signs of illness were pulled out, classified as morbid, and treated with an antimicrobial if the rectal temperature was ≥39.5°C. Antimicrobial treatments were conducted following a veterinarian's recommendation. A post-treatment interval of 3 days was implemented after the first and second treatments. If cattle remained morbid after the third treatment and the prognosis of a full recovery was unlikely, cattle were removed from the study.

**Table 1:** Composition of experimental diets (DM basis).

**Item**	**Basal diet^a^**
**Ingredient composition, % DM**	
Sudangrass hay	8.00
Alfalfa hay	4.00
Tallow	2.50
Molasses, cane	4.00
Distillers Grains w/solubles	17.50
Steam flaked corn	61.07
Urea	0.80
Dicalcium phosphate	0.15
Limestone	1.56
Magnesium oxide	0.12
TM Salt^b^	0.30
Dry matter, %	89.2
Net energy for maintenance, Mcal/kg	2.20
Net energy for gain, Mcal/kg	1.53
Crude protein, %	14.9
Rumen degradable intake protein, %	60.0
Rumen undegradable intake protein, %	40.0
Ether extract, %	7.17
Ash, %	5.89
Nonstructural CHO, %	53.9
Neutral Detergent Fiber, %	20.5
Calcium, %	0.80
Phosphorus, %	0.40
Potassium, %	0.83
Magnesium, %	0.28
Sulfur, %	0.20

^a^Basal diet supplemented with: CON: control no antibiotic; MON, supplemented with 30 mg/kg (0.018% of diet, DM basis) monensin (Rumensin 80, Elanco Animal Health, Greenfield, IN); EO+HyD: 200 mg/kg DM (0.018% of diet, DM basis) Crina^®^ (dosage 0.1g/kg D.M.) plus HYD^®^ (dosage 0.1mg/kg D.M.). Crina^®^ + (D.S.M. Nutritional Products) is a eubiotic blend composed of thymol, eugenol, limonene, and vanillin combined with HyD^®^ (25-hydroxyvitamin D3) supplemented to provide.

^b^Trace mineral salt contained: CoSO4, 0.068%; CuSO4, 1.04%; FeSO4, 3.57%; ZnO, 0.75%; MnSO4, 1.07%; KI, 0.052%; and NaCl, 93.4%.

Steer full body weight (BW) was recorded every 28 days until the end of the experiment (day 286) to monitor live weight changes. Steers were not denied feed or water before weighing. In the determination of average daily gain (ADG), interim and final weights were reduced by 4% to account for digestive tract fill (20). On June 21, 18 steers (1 steer per pen) were orally administered a SmaX-tec intraruminal boluse. SmaX-tec animal care technology^®^ enables the continuous (every 10 min) real-time display of ruminal temperature. The data were measured with the help of specific antennas (smaX-tec animal care technology^®^, Graz, Austria). From July 12 through December 6, respiration rate measures (breaths per minute) for these same steers, were observed weekly at 1,100 h (5 h following the morning feeding) by trained personnel.

### Carcass measurements

Hot carcass weights were obtained from all steers at slaughter (286 days on trial). After carcasses were chilled for 48 h, the following measurements were obtained: (1) longissimus muscle area (ribeye area), taken by direct grid reading of the muscle at the twelfth rib; (2) subcutaneous fat over the ribeye muscle at the twelfth rib taken at a location 3/4 the lateral length from the chine bone end; (3) kidney, pelvic and heart fat (KPH) as a percentage of hot carcass weight, and (4) marbling score (21). Assessment of liver scaring and liver scores were obtained from all steers at the time of slaughter.

### Estimation of dietary net energy (NE)

Daily energy gain (EG; Mcal/d) was calculated by the equation: EG = ADG^1.097^ 0.0557W^0.75^, where W is the mean shrunk B.W. (kg; (22)) Maintenance energy (EM) was calculated by the equation: EM = 0.086W ^0.75^. Dietary net energy for gain (NEg) was derived from net energy for maintenance (NEm) by the equation: NEg = 0.877 NEm – 0.41 (5). Dry matter intake (DMI) is related to energy requirements and dietary NEm according to the equation: DMI = (EM/NEm) + (EG/(0.877NEm – 0.41). From this relationship, dietary NE can be resolved by means of the quadratic formula: x = (–b – √ b2 – 4ac) / 2c, where: *x* = NEm, *a* = −0.42 EM, *b* = 0.887 EM + 0.41 DMI + EG, and *c* = −0.887 DMI (23).

### Weather measurement and temperature and humidity index (THI) estimation

Climatic variables (ambient temperature and relative humidity) were obtained every hour from an on-site weather station (California Irrigation Management Information System; Meloland Station) throughout the experimental period. The temperature humidity index (THI) was calculated using the following formula THI = (0.8 × Ta) + [(H/100) × (Ta – 14.4)] + 46.4, where Ta is air temperature (°C) and H is relative humidity (24, 25); Min = minimum; Max = maximum.

### Statistical design and analysis

The trial was analyzed as a completely random design, using pens as experimental units. Treatment effects were separated using Fisher's Least Significant Difference test. Treatment effects were considered significant when *P* ≤ 0.05 and were identified as trends when *P* > 0.05 and ≤ 0.10. (Stastistix 10, Analytical Software, Tallahassee, FL).

## Results

Treatment effects on growth performance and dietary NE are shown in Table 2. There were no treatment effects (*P* > 0.05) on initial, intermediate (112 d), and final (286 d) live weight. Holstein steers supplemented with MON or EO+HYD had similar (*P* > 0.05) ADG and DMI throughout the entire (1 – 286 d) feeding period. However, during the first 112 days on feed, steers supplemented with EO+HYD had a greater (*P* ≤ 0.05) gain to feed ratio (G/F) than cattle fed the control diet, but were not different from cattle supplemented with MON (*P* > 0.05). There were no treatment effects (*P* > 0.05) from 112 to 286 d or the overall G/F ratio.

**Table 2:** Influence of feeding an eubiotic blend of essential oils plus 25-hydroxy-vit-D3 on growth-performance of calf-fed Holstein steers.

	**Dietary treatments** ^**1**^			**SEM**
	**CON**	**MON, 30 mg/kg**	**EO+HYD, 200 mg/kg**	
**Weight, kg**				
Initial	153.8	155.7	156.3	3.3
112 d	326.4	331.2	333.8	5.4
286 d	583.5	595.9	599.4	7.8
**Average daily gain (kg)**				
1–112 d	1.55	1.58	1.59	0.031
112–286 d	1.47	1.51	1.52	0.035
1–286 d	1.50	1.54	1.55	0.021
**Dry matter intake (kg/d)**				
1–112 d	6.66	6.62	6.57	0.137
112-286 d	9.23	9.22	9.30	0.213
1-286 d	8.15	8.13	8.16	0.155
**Gain to feed ratio**				
1–112 d^2^	0.233^b^	0.239^ab^	0.244^a^	0.003
112-286 d	0.159	0.164	0.163	0.003
1-286 d	0.184	0.190	0.190	0.002
**Net energy for maintenance (NEm), Mcal/kg**				
1–112 d^2^	2.02^b^	2.07^ab^	2.10^a^	0.021
112-286 d	2.23	2.29	2.29	0.023
1-286 d^2^	2.19^b^	2.26^a^	2.26^a^	0.020
**Net energy for gain (NEg), Mcal/kg**				
1–112 d^2^	1.36^b^	1.40^ab^	1.43^a^	0.019
112-286 d	1.55	1.60	1.59	0.020
1-286 d^2^	1.51^b^	1.57^a^	1.58^a^	0.017
**Observed/expected NEm**				
1–112 d^2^	0.91^b^	0.94^ab^	0.95^a^	0.009
112-286 d	1.01	1.04	1.04	0.010
1-286 d^2^	0.99^b^	1.02^a^	1.02^a^	0.009
**Observed/Expected NEg**				
1–112 d^2^	0.89^b^	0.92^ab^	0.94^a^	0.012
112-286 d	1.01	1.05	1.05	0.013
1-286 d^2^	0.99^b^	1.03^a^	1.03^a^	0.012

^1^ Treatments: CON: control no antibiotic; MON, monensin (Rumensin 80, Elanco Animal Health, Greenfield, IN) and Crina^®^ + (D.S.M. Nutritional Products) is a eubiotic blend composed of thymol, eugenol, limonene, and vanillin combined with HyD^®^ (25-hydroxyvitamin D3) supplemented to provide 200 mg/kg DM Crina^®^ (dosage 0.1g/kg D.M.) plus HyD^®^ (dosage 0.1mg/kg D.M.).

^2^ Means in a row with different superscripts differ (P ≤ 0.05).

The greater results of G/F for EO+HYD vs. CON calves during the initial 112-d feeding period was also reflected by enhanced efficiency of dietary energy utilization (Table 2). Steers supplemented with EO+HYD had greater (*P* ≤ 0.05) estimated NEm and NEg based on cattle growth performance than cattle fed the control diet. The effects of MON supplementation on the efficiency of energy utilization were intermediate (*P* > 0.05) to that of CON and EO+HYD treatments. The overall observed vs. expected dietary NEm and NEg were greater for MON and EO+HYD than for CON. Observed NE for steers fed the Control diet was in close agreement with expected (OBS/EXP NEm and NEg were 0.99), whereas estimated dietary NE for steers supplemented with MON or EO+HYD exceeded expectations throughout the feeding period.

Treatment effects on carcass characteristics are shown in Table 3. Calf-fed Holstein steers supplemented with MON had greater fat thickness as measured over the longissimus than EO+HYD supplemented steers, and both were intermediate (*P* ≥ 0.05) to cattle fed the CON diet. There were no other treatment effects (*P* > 0.05) on KPH, longissimus area, marbling score, and retail yield. The health status of cattle in the current study, as well as liver abscess and liver scars at slaughter, were similar (*P* > 0.05) across treatments (Table 3).

**Table 3:** Influence of feeding an eubiotic blend of essential oils plus 25-hydroxy-vit-D3 on carcass characteristics and health score of calf-fed Holstein steers.

	**Dietary treatment** ^**1**^			**SEM**
	**CON**	**MON, 30 mg/kg**	**EO+HYD +, 200 mg/kg**	
Hot carcass weight, kg	360.3	362.9	370.9	6.85
Dressing percentage	61.7	60.9	61.9	0.50
Kidney, pelvic and heart fat,^3^ %	3.43	3.41	3.37	0.07
Fat thickness^2^, cm	0.76^ab^	0.85^a^	0.68^b^	0.06
Longissimus area,^3^ cm^2^	79.4	79.9	80.1	1.37
Marbling score^4^	5.40	5.88	5.71	0.26
Calculated yield grade	2.99	3.07	2.97	0.15
Pinkeye, %	13.33	7.50	6.67	5.33
Morbidity, %	6.67	10.00	6.67	6.02
Liver abscess, %	0.03	0.03	0.10	0.05
Liver abscess scars, %	20.0	17.5	30.0	6.80

^1^ Treatments: CON: control no antibiotic; MON, monensin (Rumensin 80, Elanco Animal Health, Greenfield, IN) and Crina^®^ + (DSM. Nutritional Products) is a eubiotic blend composed of thymol, eugenol, limonene, and vanillin combined with HyD^®^ (25-hydroxyvitamin D3) supplemented to provide 200 mg/kg DM Crina^®^ (dosage 0.1g/kg D.M.) plus HyD^®^ (dosage 0.1mg/kg D.M.).

^2^ Means in a row with different superscripts differ (P ≤ 0.05).

^3^ Kidney, pelvic and heart fat fat as a percentage of carcass weight.

^4^ Coded: minimum slight, 3.0, minimum small, 4.0, minimum modest, 5.0, minimum moderate, 6.0, and so on.

**Figure 1:**
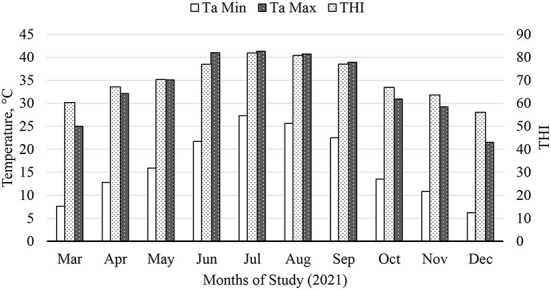
Temperature-humidity index (THI) during the course of the 286-d feeding period: THI = (0.8 × Ta) + [(H/100) × (Ta – 14.4)] + 46.4, where Ta is air temperature (°C) and H is relative humidity (Thom, 1959; NOAA, 1976); Min, minimum; Max, maximum.

**Figure 2:**
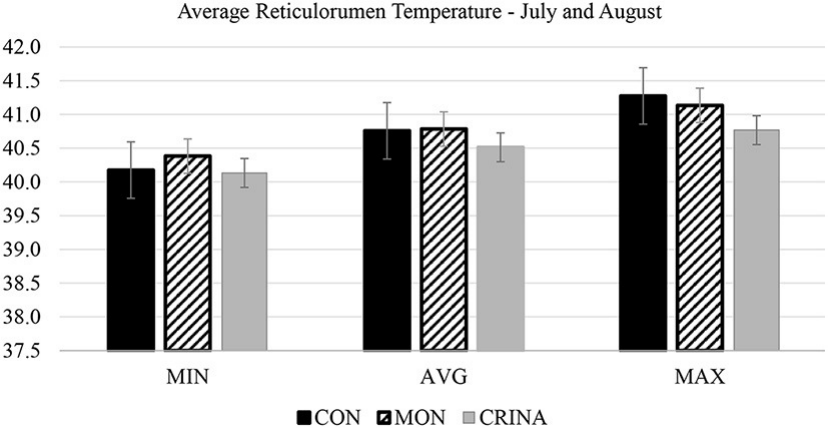
Average reticulorumen temperature during July and August.

**Figure 3:**
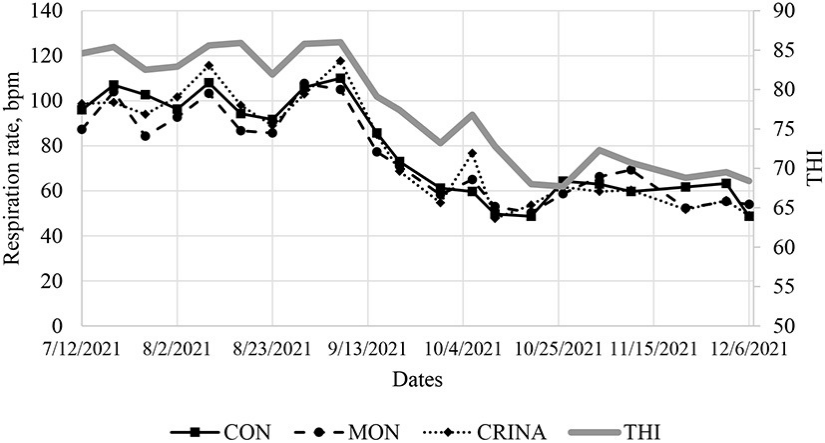
Weekly cattle respiration rate (1,100 h) and temperature-humidity index from July through December.

Monthly average THI and maximum and minimum temperature during the 286-d feeding period are presented in Figure 1. During July and August of 2021, the average THI exceeded 80, an ambient condition classified as “danger” Brown-Brandl et al. (26). Feeding EO+HYD to calf-fed Holstein steers under these ambient conditions decreased (*P* < 0.05) mean rumenal temperature compared to cattle fed CON or MON diet (Figure 2). There were no major treatment effects on cattle respiration rate (Figure 3). However, it is worth pointing out that regardless of treatment, cattle respiration rate was above “danger” designation (26) during July and August, and were highly correlated with THI measurements.

## Discussion

Consistent with the current study (Table 2), Meyer et al. (8) reported that crossbred yearling beef steers fed grain-based diets for 115 days and supplemented with a similar EO had similar overall ADG to the control non-supplmented and MON supplemented diet. However, different than the current experiemnt these authores observed that compared with non-supplemented control and EO supplemented cattle, cattle supplemented with MON decreased DMI, but greater G/F compared to non-supplemtend cattle (8). Meschiatti et al. (9) compared the effects of supplementing EO in a grain-based diet fed to Nellore bulls, and observed that supplemental MON decreased DMI but did not appreciably affect ADG or G/F when compared to diets supplemented with only an EO blend (9). In a similar study, Gouvea et al. (10) observed a similar increase in DMI for cattle supplemented with a blend of EO compared to cattle supplemented with MON, though cattle supplemented with a blend of EO were also supplemented with amylase. Moreover, Toseti et al. (27) observed similar G/F in Nellore bulls fed grain-based diets supplemented with EO or MON. However, authors reported that whereas bulls fed EO had similar DMI, both ADG and final BW were greater for bulls supplemented with EO than for bulls supplemented with MON (27).

Although, results comparing MON vs. EO have been inconsistent. In agreement with the current study (Table 2), Meschiatti et al. (9) and Gouvea et al. (10) observed that cattle fed EO or MON had similar estimated dietary energy utilization, but greater than non-supplemented cattle. Conversely to the current study, Mendoza-Cortéz et al. (28) reported that Zebu-British bulls fed a grain-based diet in high ambient temperature supplemented with EO+HYD had greater efficiency of dietary energy utilization of the diet compared with bulls supplemented with MON. Authors attributed the potential benneficts of supplementing EO+HYD to a greater DMI intake observed in cattle receiving EO+HYD compared to cattle in the MON (28). Moreover, the greater ADG observed by Mendoza-Cortéz et al. (28), could be attributed to potential effect that supplementing HYD would have on net protein retention (lean tissue growth), previous reported in the literature (14). However, in contrast with the present study, none previously mentioned studies had a negative (non-supplemented) control diet.

Consistent with similar studies, there was no effect of MON or EO on major carcass characteristcs (Table 3). In the current study dressing percentage, KPH, longissiumus area, marbling score, or yield grade, though the studies did not include a control diet with no feed additive (8–10). Meschiatti et al. (9) did not observe any difference between EO and MON treatments on the specific carcass characteristics listed, though, conversely to this study, it was reported that cattle had an increased HCW when supplemented with EO + amylase compared to MON. In contrast to similar studies, the current study reported an increase in fat thickness over the longissimus area for cattle supplemented with EO + HyD compared to MON while the control treatment was intermediary. As previously mentioned in the current manuscript, previous research has reported that cattle supplemented with HYD could have greater lean tissue growth due to greater net protein retention (14), this could be the reason for decrease in fat thickness observed in the current study. However, there were no differences among treatments in other major carcass characteristcs. Therefore, authors in the current experiment recommend that more research needs to be conducted to elucidate the effects of EO+HYD on the carcass composition of cattle.

Converse to the current study where authors did not observed treatment effects in percentage of liver abscess or liver abscess scars (Table 3). Meyer et al. (8) reported that cattle supplemented with MON + Tylosin and EO + Tylosin decrease, and cattle supplemented EO alone tended to decrease liver abscess incidence compared to the control (6.5, 8.6, 16.6, and 27.2%, respectively). Although previous research has reported that calf-fed Holstein steers have a greater incidence of liver abscess than native beef breed (29), studies conducted at our laboratory (19, 30) have reported lower incidences (< 10%) of liver abscess in calf-fed Holstein (more characteristic of the southwest desert region in the United States) than previous research. Although, previous research has reported that the majority of respiratory diseases in the feedlot occurred within the weeks days after cattle arrival (31), which may impact cattle growth performance. Therefore, potentially explanning the greater G/F efficiency observed in the first 112 days on feed for cattle supplemented with EO+HYD and MON, compared to non-supplemented cattle. There were no effect of cattle supplementation on pikeye incidence or cattle morbidity in the current study. Moreover, consistent with the present study (Figure 2), Silva et al. (32) also observed that lactating dairy cows supplemented with a blend of EO had reduced frequency of high rectal temperature and increased blood oxygenation. However, more research needs to be conducted to illustrate the mechanism of this potential benefit of EO and EO+HYD to cattle health and confort when animals are raised under high-ambient temperatures.

## Conclusion

Supplementation of calf-fed Holstein steers with MON or EO+HYD increases the overall efficiency of dietary net energy utilization for maintenance and gain (3 and 4%, respectively) without major effects on carcass characteristics or liver abscess incidence when fed to calf-fed Holstein steers for over 285 days. Moreover, cattle supplemented with EO+HYD decreased mean reticulorumen temperature when experiencing extremely high ambient temperature.

## Data availability statement

The raw data supporting the conclusions of this article will be made available by the authors, without undue reservation.

## Ethics statement

All procedures involving animal care and management were in accordance with and approved by the University of California, Davis, Animal Use and Care Committee (protocol # 18811 and 18812).

## Author contributions

All authors were involved in study design, data collection, data analysis, manuscript preparation, and approved the submitted version.

## Funding

This project was supported through the University of California Agricultural Experiment Station with Hatch funding from the USDA National Institute of Food and Agriculture (CA-D-ASC-6578-H), California Department of Food and Agriculture, and financial contributions from DSM, Inc.

## Conflict of interest

The authors declare that the research was conducted in the absence of any commercial or financial relationships that could be construed as a potential conflict of interest.

## Publisher's note

All claims expressed in this article are solely those of the authors and do not necessarily represent those of their affiliated organizations, or those of the publisher, the editors and the reviewers. Any product that may be evaluated in this article, or claim that may be made by its manufacturer, is not guaranteed or endorsed by the publisher.
